# Adults' experiences of perioperative opioid use: a mixed-methods study

**DOI:** 10.1097/PR9.0000000000001485

**Published:** 2026-08-14

**Authors:** Dalia Mohammed Aljohani, Esther M. Pogatzki-Zahn, Rosalind Adam, Patrice Forget

**Affiliations:** a Pain AND Opioids After Surgery (PANDOS) European Society of Anaesthesiology and Intensive Care (ESAIC) Research Group; b Department of Anesthesia Technology, Prince Sultan Military College of Health Sciences, Dhahran, Saudi Arabia; c Academic Primary Care, Institute of Applied Health Sciences, University of Aberdeen, Aberdeen, United Kingdom; d Department of Anesthesiology, Intensive Care and Pain Medicine, University Hospital Muenster, Muenster, Germany; e Department of Anaesthesia, Aberdeen Royal Infirmary, NHS Grampian, Aberdeen, United Kingdom; f Epidemiology Group, Institute of Applied Health Sciences, University of Aberdeen, Health Sciences Building, Foresterhill, Aberdeen, United Kingdom

**Keywords:** Perioperative pain, Opioid management, Shared decision-making, Patient-centred care, Psychosocial factors, Patient engagement

## Abstract

**Introduction:**

Managing acute postoperative pain is challenging because of its subjective nature and factors influencing it.

**Objectives:**

We explore adults' perioperative pain experiences, focusing on opioid use, shared decision making (SDM), satisfaction, and outcomes to improve patient-centred strategies.

**Methods:**

A mixed-methods study included a telephone survey conducted within the first 14 days postsurgery with the *International Pain Outcomes* (IPO) questionnaire and semi-structured interviews 3 months after surgery. Quantitative and qualitative data were integrated for a comprehensive understanding of pain management experiences.

**Results:**

Of 114 patients, 94% experienced adverse events, mainly anxiety (68%), drowsiness (67%), and helplessness (64%) after surgery. Nearly half (48%) had preoperative persistent pain, with patients spending a median of 40% of their time in severe pain postoperatively and 20% wishing for more pain relief. Despite this, 75% participated in treatment decisions, and satisfaction was high (median 9/10). Analysis of 15 interviews identified 4 themes: nobody is average (unique pain); active partner or passive participant (from empowered to passive, often misinterpreting SDM); working towards a goal (recovery and coping); and pain beyond medicine (emotional, social, spiritual). These findings show pain is highly individual, shaped by involvement and psychosocial factors, and highlight gaps in SDM understanding and use.

**Conclusion:**

Adults' pain management needs holistic, multimodal, individualised approaches with patient input, considering values, goals, and concerns beyond opioids. Clear discharge communication, ongoing care, and provider training are essential for safe recovery. Future research should explore how providers view and use SDM to overcome barriers and improve patient engagement.

## Abstract 2

Supplemental Digital Content is Available in the Text.

Adults' perioperative pain experiences highlighted individualised needs, decision-making gaps, and psychosocial distress, emphasising holistic, multimodal, patient-centred approaches to improve recovery, continuity, and pain management outcomes.

## 1. Introduction

Perioperative care affects patient satisfaction and long-term outcomes.^6,33,53^ Many surgical populations struggle with acute postoperative pain.^6,60^ Expecting severe preoperative pain is associated with higher postoperative pain intensity.^12,36,51^ Thus, unavoidable postoperative pain and patients' expectations must be managed.^12^ Effective pain management improves recovery, quality of life, decreases complications, and health care resource use.^35,53^

Pain is subjective and influenced by surgical, pharmacological factors, patients' previous experiences, coping methods, and culture.^1,4,23,25,41,51,62^ Therefore, understanding and managing postoperative pain requires considering personal and behavioural factors that affect patients' experience, interpretation, and response to pain.^1,4^ Current guidelines support multimodal and patient-centred approaches^28,33,35^; however, limited information exists on how patient involvement in pain treatment decisions affects perioperative pain management, satisfaction, and recovery. Health care providers must understand postoperative pain management from patients' perspectives to identify avenues to improve care.^4,8,11,56^

Opioids effectively treat acute pain but have various adverse effects.^29,33^ Our scoping review^4^ found that a complex interplay of individual, social, and health care–related factors influences decisions about opioid use. These included patients' understanding of opioids, emotional responses, and perceptions of benefits and risks. External factors involved the accessibility of opioids and alternative strategies, the quality of patient–provider communication, and opportunities for shared decision making (SDM). Patients tried to manage opioid intake, from distraction to controlled use, and aimed to maintain activities or avoid addiction and side effects. The review revealed a significant gap: few studies explore patients' perioperative pain management experiences, especially regarding opioid use, SDM, and satisfaction. Understanding these helps develop customised perioperative pain management approaches that support patients, improve communication, and strengthen patient–clinician relationships.

The overall aim of this study was to describe and explore adults' perioperative pain management experiences and provide insights into their perceptions at 2 time points after surgery: 14 days and 3 months. Ultimately, it aimed to guide strategies that support and improve patient-centred postoperative pain management. Specifically, the study aimed to:

(1) Describe patients' satisfaction with pain management and involvement in pain treatment decisions.
(2) Describe patients' desire for additional pain treatment and their involvement in pain treatment decisions.
(3) Explore adults' experiences and perceptions of pain medication and shared decision making, particularly regarding opioid use.
(4) Understand the influence of perioperative, individual behavioural, and external social and environmental factors on perioperative pain management.

## 2. Methods

This study used an explanatory sequential mixed-methods design, in which quantitative data were collected and analysed first, followed by qualitative data collection to further explain and contextualise the quantitative findings.^21^ Questionnaire data were collected and analysed to understand adults' perioperative pain experiences and to guide interview questions for deeper insights. Ethical approval to conduct this study was obtained from the London City and East Research Ethics Committee (ID 276689).

### 2.1. Sampling and participant recruitment

Participants were recruited from a larger epidemiological project using convenience sampling.^44^ During the quantitative phase, all willing patients from the initial study were included, offering insights to examine further.^30^ This phase was descriptive, not designed to test hypotheses; hence, a power calculation was not done.^21^ We aimed to recruit at least 100 patients, as smaller samples risk bias in quantitative pain studies.^26^ For the qualitative phase, interested patients were purposively selected to ensure diversity, providing broad variation while reflecting the wider sample.^18,45^

#### 2.1.1. Inclusion and exclusion criteria

The target population was hospitalised adult patients aged 18 years or older who were undergoing or scheduled to undergo elective or emergency surgery and received general anaesthesia, sedation, or local anaesthesia with a planned overnight stay in hospital after surgery. Exclusions: (1) American Society of Anaesthesiologists (ASA) grade V (a moribund patient not expected to survive without the operation) or VI (a declared brain-dead patient whose organs are being removed for donor purposes); (2) refusal to participate; (3) inability to give written informed consent.

#### 2.1.2. Participant recruitment

The UK Pain And Opioids After Surgery (PANDOS) study,^46^ a prospective observational cohort study, was used to identify those interested in this mixed-methods study by indicating willingness for further contact on the main consent form.

Participants from varied backgrounds were invited based on gender, age, surgical type, urgency, cancer treatments, anaesthetic type, substance use disorder (SUD), opioid use during/after surgery, and chronic pain history. Microsoft Teams Telephony was used to schedule convenient interviews, and travel costs for in-person interviews at the University of Aberdeen were reimbursed. All interviews were encrypted and transcribed by the lead investigator (DMA). Informed consent was obtained verbally for telephone data collection and in writing for in-person interviews from all participants.

#### 2.1.3. Data collection

##### 2.1.3.1. Telephone questionnaire (quantitative phase 1)

The International Pain Outcomes (IPO) Questionnaire validated by PAIN OUT was used to collect data.^50^ It has 13 items covering: pain intensity and its interference on activities, sleep, mobility, and breathing; adverse events including both physical symptoms (eg, nausea, drowsiness, itching, dizziness) and emotional or psychological symptoms (eg, anxiety and feelings of helplessness); perceptions of care including percentage of treatment received; satisfaction; desire to receive more pain treatment; participation in treatment decisions; information received about treatment options; and nonpharmacological pain relief. In addition, items to assess preoperative persistent pain status. Most items use an 11-point scale (0–10), with some binary yes/no questions, and 2 percentage items measure pain duration and relief. DMA administered the questionnaire by phone. Calls were scheduled at preferred times during the first 14 days postoperatively; day 14 represented the predefined cut-off for data collection. The 10- to 15-minute surveys were audio-recorded, and responses were entered into a REDCap database before analysis.^31^

##### 2.1.3.2. Follow-up interviews (qualitative phase 2)

The interview method was chosen for its flexibility, allowing deeper exploration of participants' experiences.^16,22^ The interview guide was based on the scoping review and IPO-Q results, reviewed with experts for feasibility and validity, and piloted (see Appendix 1, supplemental digital content, http://links.lww.com/PR9/A431). Questions explored perioperative pain management experiences and perception of care. The semi-structured interviews lasted about 40 minutes.

### 2.2. Data analysis

Quantitative results were analysed using SPSS with descriptive statistics. For variables, the mean ± SD or the median (IQR) were used based on distribution. Ordinal variables were summarised with percentages, frequencies, or median (IQR), depending on categories. One reported postoperative adverse symptom was considered an adverse event. Missing values were treated as such and not replaced.

Qualitative data were transcribed and imported into NVivo 12 to facilitate Reflexive Thematic Analysis (RTA). Braun and Clarke's (2022)^15,17^ approach allows flexible, in-depth analysis of meaning patterns, emphasising the researcher's active, reflexive role. Coding was developed inductively and evolved by DMA without inter-rater reliability, acknowledging subjectivity. Themes emerged through data engagement. An initial analysis identified categories for subsequent interviews, with a final analysis after all interviews. Following are RTA's 6 phases: (1) familiarising with data; (2) systematic coding; (3) generating initial themes; (4) developing and reviewing themes; (5) refining, defining, naming themes, and reviewing; and (6) writing the report with quotes.

## 3. Results

### 3.1. Patient characteristics

Of 186 participants, 114 completed the telephone survey from 8 UK sites, yielding a 61.3% response rate. Phase 1 recruitment ran from February to June 2024. Table 1 shows participant characteristics. Both genders almost participated equally, with 56 (49.1%) females and 58 (50.9%) males. The median age was 63 (50–74) years. Most patients underwent major surgeries (n = 77, 67.5%), and 40 patients (35%) underwent orthopaedic (nonspinal) surgery. Table 2 shows the anaesthetic techniques and perioperative analgesics used. Eighty-eight patients (77.2%) underwent surgery under general anaesthesia or sedation. In total, 79 participants (69%) received intraoperative opioids, and 92 participants (81%) received postoperative opioids.

**Table 1:** Patient characteristics (n = 114).

Variable	n (%) or median (IQR)
Gender	
Female	56 (49.1%)
Male	58 (50.9%)
Age	63 (50–74)
Current smoker	11 (9.6%)
Diabetes mellitus	14 (12.3%)
Substance use disorder	2 (1.8%)
Disorder before surgery	12 (10.5%)
Current metastatic cancer	6 (5.3%)
Severity of surgery	
Intermediate	35 (30.7%)
Major/complex	77 (67.5%)
Minor	2 (1.8%)
Type of surgery*	
Orthopaedic (nonspinal)	40 (35%)
Urology/kidney	23 (20%)
Gastrointestinal	20 (17.5%)
Obstetrics/C-section	7 (6%)
ENT/neurosurgery	6 (5%)
Breast	4 (3.5%)
Gynaecology	4 (3.5%)
Spinal orthopaedic/neurosurgery	3 (3%)
Plastics	1 (1%)
Vascular	1 (1%)
Others	5 (4%)

* Type of surgery represents surgical speciality groupings rather than specific procedural indications or diagnoses.

**Table 2:** Details of anaesthetic techniques and perioperative analgesic use (N = 114).*

	n	%
Anaesthetic technique		
General anaesthesia or sedation	88	77.2%
Local or peripheral nerve block	33	28.9%
Spinal or epidural anaesthesia	32	28.1%
Preoperative opioid use (past month)	31	27%
Intraoperative opioid use	79	69%
Postoperative analgesics		
Opioids	92	81%
Paracetamol	104	91%
Nonsteroidal anti-inflammatory drugs	24	21%

* Anaesthetic techniques and analgesic medications were not mutually exclusive; participants may have received more than one technique and/or drug.

#### 3.1.1. Quantitative results (within the first 14 days postoperative)

##### 3.1.1.1. Postoperative pain and related factors

Table 3 shows IPO-Q results. Overall, participants reported a median worst pain score of 7 (4–8) within the first 14 days after surgery and spent 40% (10%–60%) of their time in severe pain. Pain interference was highest for in-bed activities 6 (3–8) and lowest with breathing or coughing 1 (0–5). Nearly half of the participants, 55 (48%), reported preoperative persistent pain with a median pain intensity of 7 (5–9). In the supplement, Table S1 (see supplemental digital content, http://links.lww.com/PR9/A431) lists the worst pain scores for various surgeries.

**Table 3:** Pain-related patient-reported outcomes, perception of care and history of preoperative persistent pain within the first 14 days after surgery (n = 114).

Variable	
Pain-related patient-reported outcomes	
Worst pain intensity	7 (4–8)
Least pain intensity	2 (0–3)
% Time in severe pain since surgery*	40% (10%-60%)
Interference with in-bed activities	6 (3–8)
Interference with breathing or coughing	1 (0–5)
Interference with sleep	4 (1–7)
Interference with out-of-bed activities (n = 111)	5 (2–7)
Adverse events†	**108 (94.7%)**
Nausea	53 (46.5%)
Drowsiness	76 (66.7%)
Itching	52 (45.6%)
Dizziness	53 (46.5%)
Feeling anxious	77 (67.5%)
Feeling helpless	72 (63.1%)
Perceptions of care	
Overall satisfaction with pain management	9 (8–10)
Patients reporting some degree of participation in pain treatment decisions‡	85 (75%)
Degree of participation in pain treatment decisions§	8 (6–10)
Patients with a desire for more pain treatment‖	23(20%)
Patients reporting >50% pain relieved since surgery	70 (61%)
Patients reporting ≤50% pain relieved since surgery	44 (39%)
Patients reporting receipt of information about pain treatment options	62 (54%)
Patients reporting use (or receipt) of nonmedicine methods for pain relief	38 (33%)
Patients reporting preoperative persistent pain¶	55 (48%)
Intensity of preoperative persistent pain	7 (5–9)

Data are presented as median (interquartile range) for numeric rating scale items because of nonnormal distributions, and as n (%) for categorical variables. Numeric rating scales range from 0 to 10 unless otherwise stated.

* Percentage of time spent in severe pain since surgery, on a 0% to 100% scale.

† Patients who reported at least one postoperative adverse symptom.

‡ Patients who reported some degree of participation in pain treatment decision by scoring >0 to “Were you allowed to participate in decisions about your pain treatment as much as you wanted to?”

§ This is the SDM participation score (0 = not at all, 10 = completely participated in pain treatment decisions).

‖ Patients who responded “Yes” to the IPO item “Would you have liked more pain treatment than you received?

¶ Patients who responded “Yes” to “Did you have a persistent painful condition for 3 mo or more before coming for this surgery?”

Adverse events affected 108 participants (94.7%), including nausea in 53 (46.5%), drowsiness in 76 (66.7%), itching in 52 (45.6%), and dizziness in 53 (46.5%). Emotional distress was common, with 77 (67.5%) experiencing anxiety and 72 (63.1%) feeling helpless.

Supplemental digital content (see Tables S2 and S3, http://links.lww.com/PR9/A431) provide more information on adverse event severity and care perceptions.

##### 3.1.1.2. Postoperative perception of care: desired more pain treatment, shared decision-making, and satisfaction with pain treatment

As shown in Table 3, overall satisfaction with pain management was high, 9 (8–10); 85 patients (75%) reported some degree of participation in pain treatment decisions, with a median degree of 8 (6–10); yet 23 (20%) of all patients reported a desire for more pain treatments. Regarding patients' self-reported pain relief since surgery, responses were categorised as >50% and ≤50% pain relief, with 70 (61%) of patients reporting >50% relief and 44 (39%) reporting ≤50% relief.

Table 4 shows that among 85 patients (75%) reporting some degree of participation in pain treatment decision making, 72 (84.7%) reported no desire for more pain treatment, whereas 13 (15.3%) reported a desire for more relief. The median SDM score was 9 (6–10) among those without a desire for more relief and 6 (5–7) among those who did. Of 29 patients (25%) reporting no SDM participation, 19 (65.5%) reported no desire for more pain treatment, whereas 10 (34.5%) reported a desire for more relief.

**Table 4:** Desire for more pain treatment by shared decision making participation status.

Desire for more treatment	Patients reporting some degree of participation in pain treatment decisions*	Degree of participation in pain treatment decisions†	No participation in pain treatment decisions‡	Total
Yes	13 (15.3%)	6 (5–7)	10 (34.5%)	23 (20.2%)
No	72 (84.7%)	9 (6–10)	19 (65.5%)	91 (79.8%)
Total	85 (75%)	—	29 (25%)	114 (100%)

Data are presented as number (%) and median (IQR).

* Patients who reported some degree of participation in pain treatment decision by scoring >0 to “Were you allowed to participate in decisions about your pain treatment as much as you wanted to?”

† This is the SDM participation score (0 = not at all, 10 = completely participated in pain treatment decisions).

‡ Patients who reported no participation in pain treatment decision by scoring = 0 to “Were you allowed to participate in decisions about your pain treatment as much as you wanted to?”

Table 5 shows that among 85 patients (75%) reporting some degree of SDM participation, the median satisfaction with pain treatment score was 10 (8–10), whereas among patients reporting no SDM participation, the median satisfaction with pain treatment score was 7 (3.5–10).

**Table 5:** Satisfaction with pain treatment by shared decision-making participation status.

	N (%)	Satisfaction with pain treatment,* median (IQR)
Patients reporting some degree of participation in pain treatment decisions†	85 (75%)	10 (8–10)
No participation in pain treatment decisions‡	29 (25%)	7 (3.5–10)
Total	114 (100%)	

* The satisfaction scale (0 = not satisfy, 10 = extremely satisfied).

† Patients who reported some degree of participation in pain treatment decision by scoring >0 to “Were you allowed to participate in decisions about your pain treatment as much as you wanted to?”

‡ Patients who reported no participation in pain treatment decision by scoring = 0 to “Were you allowed to participate in decisions about your pain treatment as much as you wanted to?”

In Table 6, patients' perceptions of care are summarised according to whether they received intra-operative and postoperative opioids. Most patients received intra-operative opioids (79 of 113%, 70%), and 92 of 114 (81%) received postoperative opioids. Perceptions of care were broadly similar across both groups, especially regarding satisfaction with pain treatment ratings of 9 or 9.5. Whereas a minor variation in patients with a desire for more relief is noted between those who received intraoperative opioids (18%) and those who did not (26%).

**Table 6:** Perceptions of care by intraoperative and postoperative opioid exposure.

	Intraoperative opioids		Postoperative opioids	
	Yes 79 (70%)	No 34 (30%)	Yes 92(81%)	No 22(19%)
Patients with a desire for more pain treatment* Total = 23 (20%)	14 (18%)	9 (26%)	19 (21%)	4 (18%)
Shared decision making (SDM): Patients reporting some degree of participation in pain treatment decisions† Total = 85 (75%)	56 (71%)	28 (82%)	71 (77%)	14 (64%)
Degree of participation in pain treatment decisions‡	8 (6–10)	7.5 (5–10)	8 (6–10)	10 (5–10)
Satisfaction with pain treatment	9 (8–10)	9 (8–10)	9 (8–10)	9.5 (5–10)

Proportion of patients' perceptions of care: desire for more pain treatment, shared decision-making (SDM) participation, and satisfaction with pain management by intraoperative and postoperative opioid exposure. Data are presented as n (%) or as median (IQR), as indicated. Percentages are calculated after excluding missing values. SDM and satisfaction scores range from 0 to 10. Groups are not mutually exclusive, as many participants who received intraoperative opioids also received postoperative opioids.

* Patients who responded “Yes” to the IPO item “Would you have liked more pain treatment than you received?”

† Patients who reported some degree of participation in pain treatment decision by scoring >0 to “Were you allowed to participate in decisions about your pain treatment as much as you wanted to?”

‡ This is the SDM participation score (0 = not at all, 10 = completely participated in pain treatment decisions).

Supplemental digital content (see Tables S4 and S5, http://links.lww.com/PR9/A431) provide more information on care perceptions by surgery type and gender.

#### 3.1.2. Qualitative findings (at 3 months postoperative)

Fifteen of 114 participants were interviewed, including selected surgical patients from phase 1. Table 7 presents the characteristics of the 15 participants, illustrating variation in sex, age, surgical urgency, procedure type, anaesthetic technique, opioid exposure, and preoperative persistent pain. This phase ran from June to November 2024. Participants were recruited from various sites across the UK: Aberdeen Hospitals (6 patients), King George Hospital (2 patients), Hinchingbrooke Hospital (1 patient), Cornwall Hospitals (4 patients), and Stepping Hill Hospital (2 patients). Four themes identified: (1) Nobody is an average person, (2) Active partner or passive in pain management, (3) Working towards a goal, (4) Pain beyond medicines.

**Table 7:** Interview participants (n = 15).

Participant ID	Sex (age)	Surgical urgency	Surgical procedure category	Surgery for cancer treatment	Substance use disorder	Anaesthetic technique	Intravenous opioid given in surgery	Postsurgery opioids	Persistent pain
P1	M (66)	No	Laparoscopic—urology and kidney Robotic	Yes	No	General/sedation	Yes (oxycodone, remifentanil)	Yes	No
P2	M (63)	No	Open—spinal (orthopaedic or neurosurgery)	No	No	General/sedation	Yes (oxycodone, remifentanil)	Yes	Yes
P3	M (70)	No	Laparoscopic—other (including hernia repair, or intraperitoneal/retroperitoneal surgery, like oncological staging) Robotic	No	No	General/sedation	Yes (morphine, fentanyl)	Yes	No
P4	F (65)	No	Laparoscopic—other (including hernia repair, or intraperitoneal/retroperitoneal surgery, like oncological staging)	No	No	General/sedation	Yes (oxycodone)	Yes	Yes
P5	F (65)	No	Laparoscopic—upper/lower gastro-intestinal	No	No	General/sedation	Yes (oxycodone, fentanyl)	Yes	No
P6	M (67)	No	Laparoscopic—urology and kidney Robotic	Yes	No	General/sedation	Yes (oxycodone, remifentanil)	Yes	No
P7	M (30)	No	Open—upper/lower gastro-intestinal	No	No	General/sedation	Yes (oxycodone, fentanyl)	Yes	No
P8	M (61)	No	Open—orthopaedic (nonspinal)	No	No	Spinal/epidural	No	Yes	Yes
P9	M (46)	No	Open—orthopaedic (nonspinal)	No	No	General/sedation	Yes (morphine, remifentanil)	Yes	Yes
P10	F (78)	No	Open—orthopaedic (nonspinal)	No	No	Spinal/epidural and local/peripheral nerve block	No	Yes	Yes
P11	F (79)	Yes	Laparoscopic—upper/lower gastro-intestinal	Yes	No	General/sedation	Yes (oxycodone, remifentanil)	Yes	No
P12	M (80)	Yes	Laparoscopic—upper/lower gastro-intestinal	No	No	General/sedation	No	No	Yes
P13	M (79)	No	Laparoscopic—upper/lower gastro-intestinal	Yes	No	Spinal/epidural and general/sedation	Yes (fentanyl)	Yes	Yes
P14	M (68)	No	Laparoscopic—upper/lower gastro-intestinal	No	No	General/sedation and local/peripheral nerve block	Yes (oxycodone)	Yes	No
P15	F (35)	No	Open obstetrics/caesarean section	No	No	Spinal/epidural	No	No	No

This table describes characteristics of interview participants to illustrate diversity in demographic factors, surgical categories, anaesthetic techniques, opioid exposure, and preoperative persistent pain. Detailed surgical indications were not consistently available and were not the focus of the qualitative sampling.

### 3.2. Theme 1: Nobody is an average person

Participants showed individual approaches to surgery and pain management, with differences in understanding, attitudes towards opioids, and confidence in managing pain. Some noted that previous surgical experience improved their opioid knowledge.

(P2): “I would say I had a medium amount of knowledge beforehand, but my knowledge increased. I've learned about the right dosage and the right time to take medication.”

Patients' understanding of perioperative pain management influenced their experiences, especially regarding opioid risks like addiction or dependence. Concerns often stemmed from past addiction experiences.

(P11): “*I've done alcohol all my life, and I was aware that I could very easily become addicted to things.*”

Despite concerns about addiction, participants viewed opioids as safe under professional supervision, often caveating their worries.

(P3): “*It is an addictive drug but given in the right quantities and the right amount by a proper health professional, I don't see any danger in it at all.*”

Beliefs and past experiences shaped patients' responses to pain. They saw pain as subjective, with varied responses despite similar surgeries, anaesthetics, and traits. Some believed pain medication was unnecessary, trusting high pain thresholds, whereas others saw pain endurance as inherited, comparing themselves to family.

(P5): “*I have a high pain threshold because I’ve had three caesarean sections. Nothing compares to them.*”

Individuals' perceptions about opioids shaped their pain expectations, especially how they compared their preconceived expectations about pain and medication to actual postoperative experiences. Participants expected pain and worried about opioids after discharge, but some were surprised by little to no pain, influenced by past surgeries, friends' stories, and their beliefs about pain tolerance. Surgical and anaesthesia methods are key factors in whether postoperative pain occurs.

(P6): “*I expected a bit more pain because it was more intrusive than the robotic surgery.*”

### 3.3. Theme 2: Active partner or passive participant in pain management

Patients' involvement in perioperative pain management varied. Some actively participated in SDM and collaborated with providers to develop personalised plans that enhance trust and ensure effective communication. Others took a passive approach, strictly following prescribed regimens without engagement. Their choices are shaped by perceptions of pain, understanding, and medication concerns, affecting emotional and behavioural responses.

(P7): “*I would say it was very mutual [treatment decision]–– My surgical consultant always heard me out, gave clear answers, and respected me as an individual–– I was quite vocal when I was in the hospital. As to the fact that you know, my requirements for pain control could drop off*”

(P13): “*They do discuss things with you. They talked it through very carefully, and they had it [opioid] standing by if I changed my mind; I think I couldn't have asked for anything more than that.*”

Patients felt empowered by participating in their pain management decisions and understanding health care professionals, increasing comprehension. Others felt limited or “infantilised” because providers sometimes reduced their autonomy and engagement, making them passive in decision-making and undermining their confidence in the treatment plan.

(P7): “*Sometimes they don't have to infantilise the people they'*re *speaking to. I prefer kind of direct statements as to what's happening and being involved in that conversation–– sometimes a patient's opinion and feeling are disregarded*.”

Limited resources and time hindered active engagement.

(P12): “*It's obviously a desirable thing, but you know about resources, time, and everything else. It may benefit more people if the time spent in consultations between patients and professionals is reduced, allowing more time for more people. It's also the balancing act, I think, and until resources are unlimited, it's probably going to be a perpetual problem.*”

Some felt they had no choice in decisions and had to accept things as they were, leading to frustration with a passive role in pain management.

(P15): “*I was not really part of the decision on that medication. It was not like I had a choice–– I was just told that the medication was going to help me with the pain, but not in too many details like the risks and all that and not really.*”

Regardless of the adopted approaches, participants trusted their health care providers as experts. Some hesitated to engage in pain management decisions; in particular, passive recipients lacked guidance on stopping pain relief, especially when pain ceased. P(10) took opioids for about 3 months postsurgery with no pain, which led to experiencing ongoing side effects.

(P10): “*I was told how long to continue, and that was the problem–– I stopped taking it [Paracetamol and co-codamol] because I became ill–– I thought that I had to continue taking what they supplied me with until they ran out.*”

Patients with unplanned medication tapering had to decide whether to stop opioids or choose alternatives without professional help, based on personal preferences or side effects.

(P7): “*Within two to three days, I was off pain relief–– You're not quite in tune with your own body. I always feel disconnected from my body because I can't actually feel what's going on.*”

### 3.4. Theme 3: Working towards a goal

Patients' proactive involvement exceeds their passive reliance on medical professionals or pharmacology during the perioperative pain management journey. Their willingness to take control of their recovery is evident in their adherence to nonpharmacological pain management strategies, such as physiotherapy, walking, or breathing techniques, and in their proactive behaviours to achieve pain management goals, including becoming physically active, caring for the newborn, or attaining adequate pain control. Some faced barriers to staying active, like a lack of hospital support and opioid use.

(P14): “*I had no help from the physiotherapists. ––I deliberately made an effort to get moving. I made myself walk. There was nobody to be with me. –– Unfortunately, the worst part of the whole experience*”

A mother who had a caesarean section struggled with inadequate pain management and had to find medication to care for her newborn immediately.

(P15): “*Honestly, it was not enough [pain medications]. Because then I get to make plans and organise to get diclofenac on my own.*”

### 3.5. Theme 4: Pain beyond medicine

The perioperative pain management journey extends beyond hospital discharge, marking a shift from structured care to self-management. During this phase, patients' experiences changed as they assumed responsibility for pain management. Social support, faith, and health care access greatly influenced their postoperative experience. Pain care involved more than medications; adults also received emotional and practical support from family, friends, and social networks, including workplace flexibility, which helped recovery by offering reassurance, motivation, and daily assistance.

(P3): “*The best pain relief was walking to the cafe downstairs and having a cup of coffee with my family, who came to visit quite a lot with the grandkids.*”

(P13): “*I went through the whole thing without anxiety—I pray I serve the living God and I’m very content.*”

Health care support, especially responsiveness, coordination, and communication among providers, is crucial for care continuity and effective pain management.

(P12): “*It seems that one department doesn’t know what another one is doing.––There didn’t seem to be any awareness from the GP surgery that this had happened.*”

## 4. Discussion

This mixed-methods study described adults' experiences with perioperative pain management and their involvement in treatment decisions. Quantitative analysis showed that most participants reported satisfaction with their pain treatment, with a high proportion reporting involvement in decision-making and a low desire for more relief. Although many felt their pain was well managed, others reported that their needs were not fully addressed despite treatment, and those often participated less in decision making. Shared decision making participation was varied by gender, knowledge, and previous experiences, with more proportion of females reporting active involvement. Anxiety, helplessness, and drowsiness were the most common adverse outcomes.

Our findings align with evidence that satisfaction with pain treatment reflects not only being treated as informed partners in care but also the effectiveness of pain relief, the patient–provider relationship, and expectation management.^40,53,57^ Shared decision making enhances the overall patient experience and reduces opioid use without compromising pain control in obstetrics and gynaecology studies,^9,13,19,48,59^ supporting its broader applicability across surgical fields, as our data showed.

Contrasting results have been reported in orthopaedic and spinal surgery studies, where SDM or person-centred care (PCC) showed no measurable benefits on pain or satisfaction,^7^ possibly because of inconsistent or incomplete implementation. Participation in pain treatment decisions did not significantly reduce the desire for additional pain treatment,^8^ but patient-reported outcomes improved significantly. Possible contributing factors include differences in population characteristics, cultural context, SDM methods, assessment timing, surgical types, and provider communication styles.^34,37^ The psychological effects of SDM on complex emotional outcomes depend on contextual factors, as these discrepancies show.

Qualitative analysis revealed 4 themes indicating adults manage pain in highly individualised ways. Differences in knowledge, experiences, beliefs, and confidence influenced their approach to pain and opioid use, starting preoperatively and continuing after discharge. “Nobody is an average person” highlights the difficulty of treating patients as homogeneous. Recognising each person's needs and preferences is vital for personalised pain strategies. The interviews confirmed that individual behavioural factors and external environmental and social influences affected patients' pain management experiences. These factors either encouraged or discouraged individuals from participating in treatment decisions.^1,4,23,41^ Females were often engaged in treatment decisions; gender was identified as a factor in our logic model,^4^ consistent with previous findings.^24,52,55^ Knowledge was a crucial factor, derived from either hospital education or prior experience. Some adults, especially older patients^11^ or those undergoing major surgery,^32^ preferred a less active role in decision-making because of trust in professionals, paternalistic expectations, or fear of errors; still, they sought knowledge, education, and respect.

Despite high SDM scores, patients misunderstood SDM in follow-up interviews, indicating gaps between perceived and actual participations. Clearly defining health care providers' and patients' roles is essential to support meaningful participation and patient-centred care.^62^ Active recipients felt empowered, confident, and informed, which built trust and collaboration with providers by respecting them as individuals.^20,38,39,41,47,56^ Conversely, those adopting more passive roles because of communication barriers, or time-limited consultations, felt undertreated, disregarded, and uncertain about their medication plans (especially regarding opioids).^32,54,61^ Targeted strategies are needed to address barriers to SDM implementation in postoperative pain management.

Insufficient education on surgical pain management and opioids leads to poor pain control, anxiety, opioid misuse, and inadequate discharge preparation.^5,12,40,63^ Comprehensive preoperative education for patients and families reduces anxiety and promotes realistic expectations that pain is inevitable, reducing opioid reliance and possibly allowing patients to forgo opioids.^1,29,36^ Interestingly, opioid knowledge was associated with prior direct or indirect exposure rather than education or employment status,^63^ highlighting the need for experience-informed education strategies, not demographics.

Patients link pain relief with functional recovery and quality of life.^43,49,64^ Despite efforts such as physical activity, physiotherapy, and medication, adverse effects, care discontinuity, and systemic barriers frequently undermined these goals. Although many interventions attempt to address these challenges and support patient-centred, goal-oriented perioperative pain management,^6,33,35,42^ similar obstacles were previously identified.^2,5,14,64^ Health care providers should support patients to achieve optimal outcomes.^40^

Despite inconsistent evidence concerning nonpharmacological methods, including faith-based coping,^3,10,25,27,64^ strong social support can improve perioperative experience, reduce pain, and emotional distress,^27,41,64^ especially during the shift from hospital-based treatment to self-management. However, participants felt unprepared or abandoned after discharge because of fragmented services and inadequate follow-up support,^58,64^ emphasising the need for more coordinated postoperative pain care that goes beyond hospital walls.

### 4.1. Strengths and limitations

This mixed-methods study is the first to explore adults' perioperative pain management at 2 postoperative time points, across surgeries, demographics, and anaesthetic techniques, including participants with preoperative persistent pain. The specific contribution lies in its explanatory sequential design that strengthened interpretation by linking quantitative and qualitative findings.^22,35,56^ It provides a patient-voiced evidence of SDM gaps, which inform patient-centred clinical practice and intervention design. However, the small convenience sample limited subgroups' significance judgment, which the PANDOS study addressed. The IPO-Q was completed at different times throughout the first 14 postoperative days, reflecting real-world follow-up, but potentially influencing reported pain outcomes. The inclusion of multiple surgical procedures and 8 UK sites introduced heterogeneity in pain trajectories and analgesic practices; while limiting surgery-specific interpretation, it strengthens external validity.

Engaging adults in perioperative pain management is key to patient-centred care. Relying solely on opioids is insufficient; care should reflect patients' values and goals, with clear communication, trust, and roles, multimodal approaches, including personalised plans that support recovery and daily functioning. Future studies should explore health care providers' perspectives on SDM, identify barriers and facilitators, and test interventions like decision aids, structured preoperative discussions, and follow-ups to enhance opioid safety, reduce distress, and bridge the participation gap. Research should investigate socioeconomic factors influencing engagement and outcomes, and extend to adolescent and paediatric populations, including parent/caregiver perspectives, to promote equitable, personalised perioperative pain management.

Despite high satisfaction and involvement in perioperative pain management, qualitative insights revealed persistent challenges with SDM, pain management, recovery, and communication. SDM misunderstanding showed a discrepancy between perceived and real participation. Perceptions of care varied by gender, prior experience, knowledge, and provider interactions. Patients ranged from empowered to passive, with recovery affected by social support, prior experience, and care coordination during the hospital-to-home transition. Perioperative opioid use alone does not meet all adult pain needs because they still have pain and concerns. Our findings emphasise perioperative pain management partnership through patient-centred approaches, shared decision making, and empowerment for safer, more effective recovery.

## Disclosures

DMA is funded by Prince Sultan Military College of Health Sciences, Dhahran, Saudi Arabia and the Saudi Arabian Cultural Bureau in London, UK. This study is part of DMA’s PhD study at the University of Aberdeen. P.F. received advisory board/speaker fees from Oncomfort, Grunenthal and GE Healthcare. E.M.P.-Z. received financial support from Grünenthal, Germany, for research activities and advisory and lecture fees from Grünenthal, Germany, MSD/MERCK, Germany, and Medtronic, Switzerland. In addition, she receives scientific support from the German Research Foundation (DFG), the Federal Ministry of Education and Research (BMBF), the Federal Joint Committee (G-BA), and the Innovative Medicines Initiative 2 Joint Undertaking under grant agreement No. 777500. This Joint Undertaking receives support from the European Union's Horizon 2020 research and innovation program and EFPIA. All money went to the institutions (WWU/UKM) E.M.P.-Z. is working for. The remaining authors have no conflict of interest to declare.

## Supplemental digital content

Supplemental digital content associated with this article can be found online at http://links.lww.com/PR9/A431.
